# Chromosome-level genome assembly of the European green woodpecker *Picus viridis*

**DOI:** 10.1093/g3journal/jkae042

**Published:** 2024-03-27

**Authors:** Thomas Forest, Guillaume Achaz, Martial Marbouty, Amaury Bignaud, Agnès Thierry, Romain Koszul, Marine Milhes, Joanna Lledo, Jean-Marc Pons, Jérôme Fuchs

**Affiliations:** Éco-anthropologie, Muséum national d’Histoire naturelle, CNRS UMR 7206, 75005 Paris, France; CIRB, Collège de France, Université PSL, CNRS, INSERM, 75005 Paris, France; Institut de Systématique Evolution Biodiversité, Muséum national d’Histoire naturelle CNRS SU EPHE UA, CP 51, 75005 Paris, France; CIRB, Collège de France, Université PSL, CNRS, INSERM, 75005 Paris, France; Université Paris-Cité, 75006 Paris, France; Institut Pasteur, CNRS UMR 3525, Université Paris Cité, Unité Régulation Spatiale des Génomes, 75015 Paris, France; Institut Pasteur, CNRS UMR 3525, Université Paris Cité, Unité Régulation Spatiale des Génomes, 75015 Paris, France; Institut Pasteur, CNRS UMR 3525, Université Paris Cité, Unité Régulation Spatiale des Génomes, 75015 Paris, France; Institut Pasteur, CNRS UMR 3525, Université Paris Cité, Unité Régulation Spatiale des Génomes, 75015 Paris, France; PlaGe, INRAE, Genotoul, 31320 Castanet-Tolosan, France; PlaGe, INRAE, Genotoul, 31320 Castanet-Tolosan, France; Institut de Systématique Evolution Biodiversité, Muséum national d’Histoire naturelle CNRS SU EPHE UA, CP 51, 75005 Paris, France; Institut de Systématique Evolution Biodiversité, Muséum national d’Histoire naturelle CNRS SU EPHE UA, CP 51, 75005 Paris, France

**Keywords:** *Picus viridis*, woodpeckers, genome assembly

## Abstract

The European green woodpecker, *Picus viridis*, is a widely distributed species found in the Western Palearctic region. Here, we assembled a highly contiguous genome assembly for this species using a combination of short- and long-read sequencing and scaffolded with chromatin conformation capture (Hi-C). The final genome assembly was 1.28 Gb and features a scaffold N50 of 37 Mb and a scaffold L50 of 39.165 Mb. The assembly incorporates 89.4% of the genes identified in birds in OrthoDB. Gene and repetitive content annotation on the assembly detected 15,805 genes and a ∼30.1% occurrence of repetitive elements, respectively. Analysis of synteny demonstrates the fragmented nature of the *P. viridis* genome when compared to the chicken (*Gallus gallus*). The assembly and annotations produced in this study will certainly help for further research into the genomics of *P. viridis* and the comparative evolution of woodpeckers. Five historical and seven contemporary samples have been resequenced and may give insights on the population history of this species.

## Teaser

The European Green Woodpecker, *Picus viridis*, is found in the Western Palearctic region. Here, Forest et al. assemble a highly contiguous genome assembly for this species using a combination of short- and long-read sequencing and scaffolded with chromatin conformation capture (Hi-C). The assembly and annotations produced in this study will help further research into the genomics of *P. viridis* and the comparative evolution of woodpeckers.

## Introduction

The European green woodpecker (*Picus viridis* Linnaeus 1758) is a common Western Palearctic species that can be found in all sort of wooded environment where it often forages on ants on the ground. This species was previously considered to be conspecific with the Iberian green woodpecker (*Picus sharpei*) and Levaillant's woodpecker (*Picus vaillantii*) but recent molecular studies suggest that these taxa may consist of distinct biological species (Perktas *et al*. 2011; Pons *et al*. 2011, 2019). European green woodpecker and Iberian green woodpecker form a secondary contact zone in southern France where individuals from the two species sometimes hybridize in the departments of Pyrénées-Orientales, Aude, Hérault and Gard, with very limited introgression outside of the contact zone (Pons *et al*. 2019). The European green woodpecker is currently considered as a common species (least concern) with a global increasing population size of 1.2–2.3 million individuals (Birdlife International 2024), among which 300,000–600,000 are thought to occur in France (Issa et al. 2015). The French population size is also considered to be increasing (+50% over the 1980–2012 time period, +2% over the 2001–2015 time period) (Issa et al. 2015) and currently represents a stronghold of this species (25% of the total population).

Here, we present a chromosome level assembly of the European green woodpecker genome, as well as genome resequencing data for another 11 individuals sampled over the 1970–2020 time period. Our assembly is based on a combination of Illumina short reads, Nanopore PromethION long reads and chromatin conformation capture (Hi-C). The vast majority of the in silico annotated genes are located on 47 chromosomal-level sequences, in accordance with the described karyotype of the species (2*n* = 94, Hammar 1970).

## Material and methods

### Sampling scheme

We sampled 13 *Picus viridis viridi*s individuals, 5 *historical* individuals collected between 1970 and 1977, and 8 *contemporary* individuals collected between 2016 and 2021 (Table 1). Toe pads sampling for the historical specimens was performed using gloves and sterile scalpel blades that were changed between each individual. Tissues for contemporary specimens consisted of muscle or heart are either preserved in ethanol or flash frozen. For the two specimens used to assemble the reference genome, the tissues consisted of heart, muscle, and liver (Nanopore long reads and Illumina short reads, MNHN ZO-2020-125) or liver (Hi-C, MNHN ZO-2021-2133) sampled 1–2 h after the individual's death and either immediately extracted (heart) or flash frozen at −80°C (liver). The two individuals originated from localities distant of 25 km from each other and are both around 800 km away from the secondary contact zone with its sister species *Picus sharpei* (Pons *et al*. 2019).

**Table 1.** Information of the sequenced individuals.

Specimen voucher	Date	Locality	Latitude	Longitude	Sex	Use
Historical						
ZO-1971-1090	1970 Nov. 16	Gif-sur-Yvette, Essonne, Ile-de-France, France	48.7	2.13	M	Resequencing
ZO-1971-1091	1971 Jul. 28	Baugé, Maine-et-Loire, Pays-de-la-Loire	47.55	−0.11	M	Resequencing
ZO-1991-185	1972	Chamonix-Mont-Blanc, Haute-Savoie, Auvergne-Rhône-Alpes	45.93	6.93	M	Resequencing
ZO-1991-186	1978	Saclay, Essonne, Ile-de-France	48.74	2.17	F	Resequencing
ZO-1993-190	1977 Apr. 10	Massérac, Loire-Atlantique, Pays-de-la-Loire	47.67	−1.91	M	Resequencing
Contemporary						
ZO-2017-195	2016 Jun. 7	Vignely, Seine-et-Marne, Ile-de-France	48.927248	2.805190	F	Resequencing
ZO-2017-257	2016 May 30	Évry-Grégy-sur-Yerre, Seine-et-Marne, Ile-de-France	48.668917	2.651980	F	Resequencing
ZO-2017-316	2016 Jun. 27	Hagenthal-Le-Bas, Haut-Rhin,Grand-Est	47.530303	7.482225	M	Resequencing
ZO-2017-334	2015 Jun. 10	Cavaillon, Vaucluse, Provence-Alpes-Côte-d’Azur	43.836603	5.040671	F	Resequencing
ZO-2018-033	2016 Jun. 15	Niherne, Indre, Centre-Val-de-Loire	46.772839	1.526087	M	Resequencing
ZO-2018-044	2016 Jul. 18	Blavozy, Haute-Loire, Auvergne-Rhône-Alpes	45.062158	3.972074	M	Resequencing
ZO-2020-125	2020 Sep. 1	Courquetaine, Seine-et-Marne, Ile-de-France	48.680485	2.749837	M	Reference Genome (short/long reads)
ZO-2021-2133 (fluid)	2021 Jul. 12	Serris, Seine-et-Marne, Ile-de-France	48.857335	2.807369	M	Reference genome (Hi-C)

All individuals were sampled in France.

### Extraction protocol

DNA was extracted using CTAB (resequencing, Winnepenninckx *et al*. 1993) or Phenol–Chloroform (reference genome) protocols. Historical specimens were extracted before any modern samples were processed. DNA quality and purity was assessed using a Qubit Quantification (Qubit dsDNA BR Assay Kit, Life Technologies), NanoDrop spectrophotometer (NanoDrop Technologies, Inc., Wilmington, DE) and Fragment Analyzer (Agilent) to assess DNA quality and quantity.

### Reference genome sequencing

DNA-seq libraries have been prepared according to Illumina's protocols using the Illumina TruSeq Nano DNA HT Library Prep Kit. Briefly, DNA was fragmented by sonication, size selection (average insert size: 400 bp) was performed using SPB beads (kit beads) and adaptators were ligated to be sequenced. Library quality was assessed using an Advanced Analytical Fragment Analyzer (Advanced Analytical Technologies, Inc., Iowa, USA) and libraries were quantified by qPCR using the Kapa Library Quantification Kit. DNA-sequencing experiments have been performed on a NovaSeq SP lane (Illumina, CA, USA) using a paired-end read length of 2 × 150 pb with the Illumina NovaSeq Reagent Kits.

Long-read library preparation was performed according to the manufacturer's instructions “1D gDNA selecting for long reads (SQK-LSK109)”. At each step, DNA was quantified using the Qubit dsDNA HS Assay Kit (Life Technologies). DNA purity was tested using the nanodrop (Thermofisher) and size distribution and degradation assessed using the Fragment analyzer (Agilent) High Sensitivity Large Fragment 50 kb Kit. Purification steps were performed using AMPure XP beads (Beckman Coulter). For one simple library, 10 µg of DNA was purified then sheared at 25 kb using the megaruptor system (diagenode). A one-step DNA damage repair + END-repair + dA tail of double-stranded DNA fragments was performed on 2 µg of sample before ligating adapters to the library. Library was loaded onto one R9.4.1 flowcell at 20 fmol then reloaded once at 11 fmol on GridION instrument within 72H. For one optimized library, 20 µg of DNA was purified then sheared at 20 kb using the megaruptor system (diagenode). A size selection step at 5 kb using Short Read Eliminator XS Kit (Circulomics) was performed on 15 µg of sample. For 5 µg of DNA, an extra repair step with SMRTbell DAMAGE REPAIR KIT (PACBIO, 100-465-900) was performed before the one-step DNA damage repair + END-repair + dA tail of double-stranded DNA fragments and adapters ligation. Library was loaded onto one R9.4.1 flowcell at 20 fmol then reloaded 3 times at 20 fmol on a PromethION instrument within 72H. All short- and long-read libraries were prepared and sequenced at the GeT-PlaGe core facility, INRAE, Toulouse.

### Hi-C

Liver frozen tissue was directly put into 50 ml of formaldehyde (3%) and phosphate buffered saline (PBS) solution. Fixation was then incubated for 1 h under gentle agitation. Glycine was added to a final concentration of 0.125 mM and the reaction was incubated for another 20 min. Tissue was recovered by centrifugation (5,000*g* for 20 min) and washed with PBS 1X before being recentrifuged. The Supernatant was discarded and tissue was stored at −80°C until use. The Hi-C library was constructed from liver tissue starting from a mass of 20 mg. Tissue was first resuspended in 1.2 ml of TE 1X and disrupted using CK14 glass beads (Precellys, Bertin Technology) and a precellys apparatus (program: 5 × 30 s ON—30 s OFF—8700 rpm). The lysate was recovered and then used as input for the ARIMA-HiC preparation kit (Arima Genomics). Libraries were then processed for sequencing as previously described (Moreau *et al*. 2018) and sequenced on a Novaseq apparatus.

### Resequencing of historical and contemporary samples

Resequencing of the 11 individuals was performed at the “Institut du Cerveau” (ICM, Pitié-Salpêtrière Hospital, Paris) using a target 250–300 bp insert size on a NovaSeq 6000 system sequencer.

### Genome reference assembly and quality assessment

A first assembly was made using the Nanopore long reads using the Flye (v2.8.1-b1676) de novo assembler (Kolmogorov *et al*. 2019). The assembly was then polished using 3 iterations of PILON (Walker *et al*. 2014), then once with Racon (Vaser *et al*. 2017), finished by a last iteration of PILON. The resulting polished assembly has been used as a guide for the Hi-C scaffolding process using instaGRAAL (Baudry *et al*. 2020).

### Genome size completeness, estimates of genome size using k-mer (SGA preqc)

SGA preqc tool (https://raw.githubusercontent.com/jts/sga/master/src/SGA/preqc.cpp) was used to do a k-mer analysis in order to estimate genome size completeness. K-mers, short DNA sequences of fixed length, were analyzed for their abundance in the genomic data. By calculating the occurrence of k-mers, it aims to make predictions about the size and completeness of the genome. It gives a hint on the quality of the data and anticipates the difficulty of the assembly process.

### K-mer composition of the genome assembly

To analyze the quality and composition of our genome assembly, we employed KAT (K-mer Analysis Toolkit) (Mapleson *et al*. 2017). This toolkit is designed for reference-free quality control of whole genome shotgun reads and de novo genome assemblies. It utilizes k-mer frequencies and GC composition to assess errors, bias, and contamination throughout the assembly process. By comparing the k-mers present in the input reads and the resulting assemblies, it offers valuable insights into the composition and quality of genome assemblies.

### Masking repeated elements

We used RepeatMasker (v4.1.2-p1) (Smit *et al*. 2013) on the genome assembly obtained from the instaGRAAL step. This program screens DNA sequences for interspersed repeats and low complexity DNA sequences. It annotates the identified repeats and hard-masks them by replacing them with Ns.

### Genome annotation

The gene structure annotation was performed using BRAKER (Hoff *et al*. 2016), an automated method that utilizes both genomic and RNA-Seq data to generate comprehensive gene structure annotations in novel genomes. Using BRAKER-2.1.6, GeneMark (Lomsadze *et al*. 2005, 2014) is first executed on a set of proteins coming from the reference proteome of *Gallus gallus* from UniProt (UP000000539). GeneMark is trained using these protein-coding sequences and produces a set of sequences for the ab initio methods used afterwards, like AUGUSTUS (Stanke *et al*. 2006), that produces gene and features predictions from the given *P. viridis* genome. The resulting gene set consists of genes strongly supported by extrinsic evidence. Some statistics on the produced annotation have been generated using AGAT (v1.2.0) (Dainat 2023).

### Genome synteny

The masked genome was globally aligned with the genome of the chicken, *G. gallus*, the model organism, using MUMmer (Kurtz *et al*. 2004). We based our methodology for analyzing synteny on the one used for the reference genome assembly of *Colaptes auratus* (Hruska and Manthey 2021). Only regions with more than 70% identity were retained, with lengths greater than 500 bp. Next, the *P. viridis* chromosomes showing the greatest overlap with chicken chromosomes were associated and renamed according to their corresponding chicken chromosome name. A synteny circos plot produced with OmicCircos (v1.36) (Hu *et al*. 2014) shows the overall chromosome rearrangement between the two species. Only chromosomes sharing at least 500 unique links were conserved in the figure. To reorder chromosomes, the median position of hits in chickens for each segment from the *P. viridis* chromosomes was used to sort them. The same approach was performed against the genome of the Northern Flicker, *C. auratus*, retaining fragments aligned with MUMmer (Kurtz *et al*. 2004) that exceed 10 kb in length and exhibit 80% identity due to the significant noise resulting from close proximity and an abundance of repeated sequences.

### Assessing assembly quality

In order to estimate the quality we used BUSCO v4.1.4 to determine the proportion of genes expected in birds, using the Aves_ODB10 dataset, i.e. the 8,338 single-copy orthologous genes cataloged for the Aves class by the OrthoDB (v10) database (Simão *et al*. 2015).

### Ultraconserved elements

We also assessed genome completeness by estimating the number of ultraconserved elements (UCEs), a set of loci commonly used in phylogenetics. We retrieved the UCEs from 13 Piciformes genomes published on Gebank (cutoff date 2022 Oct 12; Supplementary Table 2). We extracted the UCEs with *Phyluce* (Faircloth 2016) according to the online tutorial (https://phyluce.readthedocs.io/en/latest/tutorials/tutorial-3.html). The UCEs from the 14 individuals were then aligned using MAFFT (Katoh *et al*. 2002) and internally trimmed with Gblocks (Castresana 2000; Talavera and Castresana 2007). We did not allow missing loci for a species. We performed a concatenated maximum likelihood analysis with the UCE loci under the GTRGAMMA model in RAXML (Stamatakis 2014).

### Mitochondrial genome assembly

We used NOVOPlasty-2.7.2 (Dierckxsens *et al.* 2017) to assemble the mitochondrial genomes using the short reads. We specified a genome range of 16,500–19,000 bp, a K-mer size of *k* = 39 and an insert size of 300 bp. As a seed, we used an ATP6 sequence from another *P. viridis viridis* individual (MF766578, Shakya *et al*. 2017). When the genome was not circularized, we used the BWA algorithm, as implemented in Ugene (Okonechnikov *et al*. 2012) using the default option except the number of differences that we set to 4.

We performed a mitochondrial genome analysis using all Piciformes sequences that are available on Genbank (cutoff date 2023 Jul 15). We restricted our analyses to 12 protein-coding genes (we did not include ND6 as it was not present in several genbank sequences). We used the mitochondrial DNA genome of *Galbula dea* as an outgroup (MN356220) and included 36 other Piciformes (35 individuals available on Genbank and the newly produced sequence of *P. viridis*) (Supplementary Table 1). We did not include *Yungipicus canicapillus* MK335534 because it is a chimeric sequence between *Y. canicapillus* and *Dendrocopos darjellensis* (Fuchs et al., in preparation). Stop codons were excluded from the alignments as well as the extra nucleotide found in position 174 in ND3 in most Piciformes species. Substitution models were selected under the Bayesian Information Criterion, as implemented in MEGA X (Kumar *et al*. 2018). Singe locus and concatenated partitioned maximum likelihood analyses were performed using RAXML on the RAXML Blackbox (Kozlov *et al*. 2019). Clade robustness was estimated using 100 bootstrap replicates.

### Population resequencing analysis and variant calling

From the newly assembled reference genome, we used snpArcher (Mirchandani *et al*. 2024) to call variants. This pipeline involves a mapping of all the reads on the reference genome and for those where the coverage and depth are sufficient, it marks each position that differs from the reference and merge all individuals into a single file in VCF format (Variant Call Format). We computed the number of segregating sites, S, and Watterson's Θ, using PopGenome 2.7.5 (Pfeifer *et al*. 2014) and the Nei diversity index π (Nei and Li 1979) using VCFtools (Danecek *et al*. 2011) (Table 2). Principal component analysis (PCA) were produced using pcadapt (v4.3.3) (Privé *et al*. 2020). Hierarchical clustering analysis was performed on a dissimilarity matrix of all the samples using SNPRelate (v1.32.2) (Zheng *et al*. 2012). snpArcher automatically applies specific filters using the HaplotypeCaller from the genome analysis toolkit (GATK) to customize our approach to the dataset’s characteristics. To heighten sensitivity and include variants in areas with inadequate read support, we set the –min-pruning filter to a permissive threshold of 1, meaning that variants with as little as one supporting read are considered for inclusion in the final variant call set. Additionally, the –min-dangling-branch-length filter is set to a value of 1, enabling even short “dangling branches” in the assembly graph to be conserved and considering variants with minimal support. Nevertheless, it is essential to recognize that these filter settings boost sensitivity while potentially increasing the probability of false positive variant calls. These parameter selections were made carefully to achieve an equilibrium between sensitivity and specificity while considering the characteristics of our dataset. This has been carried out for subsequent filtering, after the construction of VCF, to enable fine-tuning of filters according to the observed depth distribution. Following these primary filters, further filtering based on coverage depth (DP) is applied to each variant, after the application of GATK caller filters. After implementing the initial filters, each variant undergoes depth-based filtering based on the depth of coverage (DP) once the GATK caller filters have been applied. Each variant is retained only if the depth exceeds 10× and falls below the 90th percentile of the observed depth distribution, in order to eliminate outliers.

**Table 2.** Summary statistics for the resequencing nuclear data, as estimated by PopGenome (Pfeifer *et al*. 2014) and VCFtools (Danecek *et al*. 2011).

	Total	Autosomes	Z chromosome
Number of segregating sites (S)	2,407,525	2,103,265	304,260
Normalized segregating sites by sequence length (s)	0.001	0.001	0.002
Nei diversity index (π)	0.0009	0.0009	0.0008
Watterson's estimator per site (Θ)	0.0005	0.0004	0.0007
Historical individuals			
Number of segregating sites (S)	1,606,510	1,398,591	207,919
Normalized segregating sites by sequence length (s)	0.001	0.001	0.001
Nei diversity index (π)	0.0011	0.0011	0.0010
Watterson's estimator per site (Θ)	0.0004	0.0004	0.0006
Contemporary individuals			
Number of segregating sites (S)	1,606,510	990,064	145,932
Normalized segregating sites by sequence length (s)	0.001	0.0008	0.001
Nei diversity index (π)	0.0008	0.0009	0.0007
Watterson's estimator per site (Θ)	0.0004	0.0002	0.0004

### Site frequency spectrum

The site frequency spectrum (SFS) represents the distribution of the allelic frequencies of the mutations throughout the genome (Fu 1995). It gives the number of mutations present at each frequency. The folded SFS of a sample of *n* diploid individuals is described as a vector η such that η = (η_1_, η_2_,…, η_2*n*−1_), where η*_i_* is the number of mutations at frequency *i*/2*n* with *i*∈[1:2*_n_* − 1]. Spectra are also normalized and transformed to ϕ *_i_* = η*_i_* × *i*(*n* − *I*)/Ση*_i_*, except for *i* = *n*, where ϕ *_i_* = *n*/2Ση*_i_* (Achaz 2009).

## Results

### Reference genome sequencing

Heart provided less-degraded DNA when compared to pectoral muscle or liver for long-read sequencing. We obtained 41.2 Gbp of long reads (Gridion: 3.5 Gbp, N50 close to 16 kb; Promethion: 37.7 Gbp, N50 close to 10 kb) as well as about 65 Gbp of short-read data. The SGA preqc genome size estimate for *P. viridis* was 1.26 Gb. From mapping information, coverage depth is around 73× (Supplementary Table 5).

### Assembly quality and genome completness

**Fig. 1.**
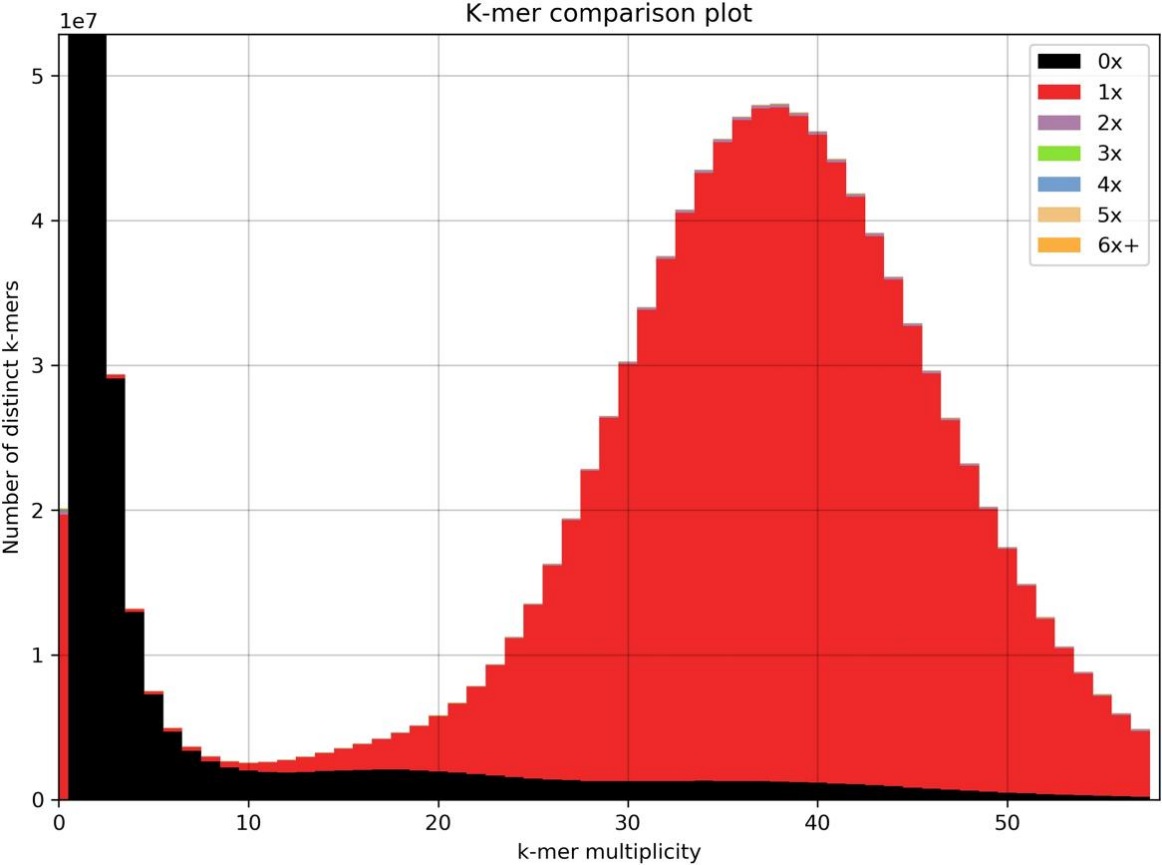
Distribution of k-mers using KAT. In the k-mer spectrum, reads that are absent from the assembly are displayed in black. The error distribution is quite low with most of errors under 10×.

The majority of the assembly presents unique k-mer content that is only present once (Fig. 1). This pattern is typical of what we expect from a complete haploid assembly, generated from a diploid genome.

Results from the BUSCO analyses indicated that 93.6% of the loci were included in the assembly (single-copy: 88.7%, duplicated: 0.4%, fragmented: 4.5%). Comparison with other Picidae genomes indicated our genome assembly ranks third out of five for genome completeness (Supplementary Table 2) and third out of four for genomes for which long reads were generated.

**Fig. 2.**
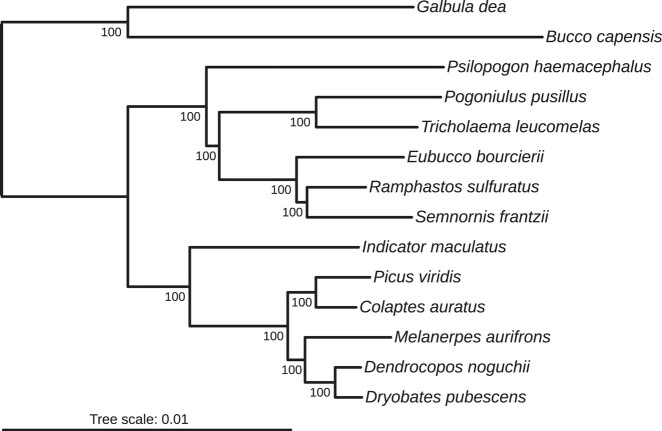
Phylogenetic tree showing relationships among Piciformes species resulting from maximum likelihood analysis of Ultra Conserved Elements loci. The tree was reconstructed from an alignment of 631,268 nucleotides (2,906 loci). The bootstrap percentage (BP) is indicated on the nodes.

The second highest number of retrieved UCEs in the Piciformes was found in our *P. viridis* assembly, confirming its high completeness (Supplementary Table 3). Alignment of the concatenated UCEs (2906 loci) was 631,268 bp long, among which 16,077 sites were informative. The result of the maximum likelihood analyses recovered the same topology (Fig. 2) at the family level than Prum *et al*. (2015), with all nodes being supported by bootstrap values of 100.

### Mitogenome assembly

Our final assembly of the mitochondrial genome was 16,912 bp long, with some uncertainty concerning the exact length due to the presence of a 64 bp repeat at the end of the first control region as well as one C monomeric region in 16S (Supplementary Table 4). The genome is similar to many other avian genomes with 13 protein-coding genes, 22 transfer RNA and 2 Ribosomal RNA. The control region is duplicated, presenting 1 functional control region and 1 degenerated control region.

**Fig. 3.**
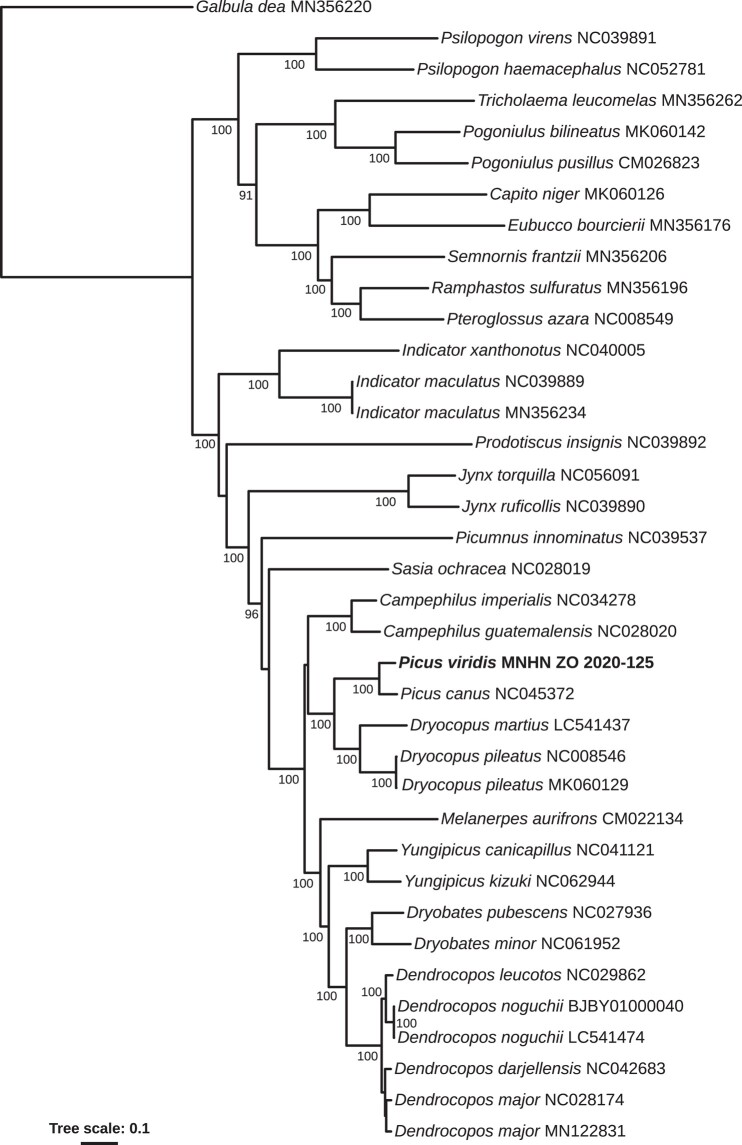
Maximum likelihood phylogenetic tree showing relationships among Piciformes species. Twelve mitochondrial loci, 37 taxa, and 10,857 characters were included in the analysis. The bootstrap percentage (BP) is indicated on the nodes.

The topology recovered from the partitioned concatenated analysis of 12 mitochondrial protein-coding loci (Fig. 3) was in strong agreement with current phylogenetic hypotheses for family-level and genus-level relationships in Piciformes (e.g. Prum *et al*. 2015; Shakya *et al*. 2017).

### Genome annotation

A total of 15,848 protein-coding genes were identified, representing 81,357,234 bp in total, along with essential transcription factors, noncoding RNAs, and repetitive elements, through automated genome annotation; 16,212 mRNA, representing 82,809,862 bp in total, were identified, including 364 isoforms; 77,423 exons are numbered, at a rate of 4.8 exons per coding DNA sequence (Supplementary Table 9).

### Repetitive elements

Detailed analysis of repetitive elements in the *P. viridis* genome revealed their abundance, comprising 30.1% of the assembled genome (385,325,029 out of 1,279,164,199 total bp). This estimate compares with the 28% recovered for *C. auratus* (Hruska and Manthey 2021), 25.8% for *Melanerpes aurifrons* (Wiley and Miller 2020), and 22% for *Dryobates pubescens* (Zhang *et al*. 2014). Details of the types of repeated elements found using Repeatmasker can be found in Supplementary Table 8.

### Genome synteny

**Fig. 4.**
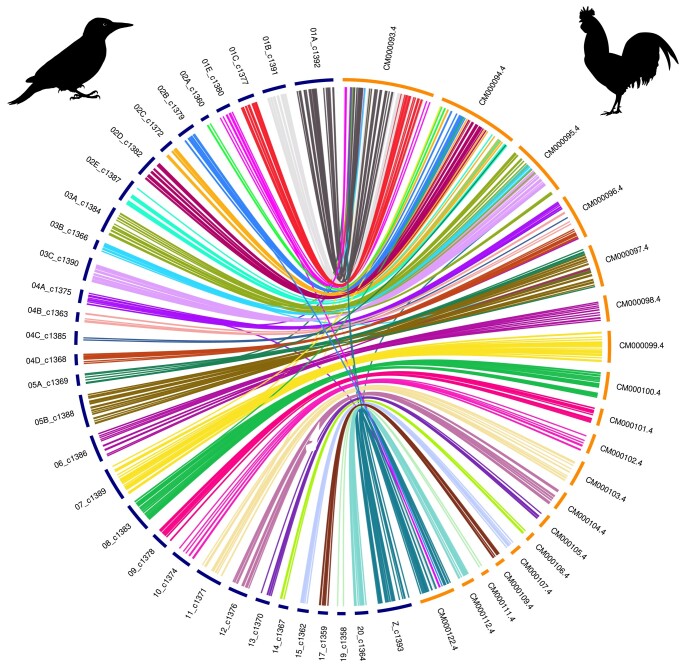
Circos plot. Woodpecker chromosomes are on the left, in blue; chicken chromosomes are on the right, in yellow. Note that the number of chromosomes differs between the Chicken *Gallus gallus* and the European green woodpecker *Picus viridis.*

**Fig. 5.**
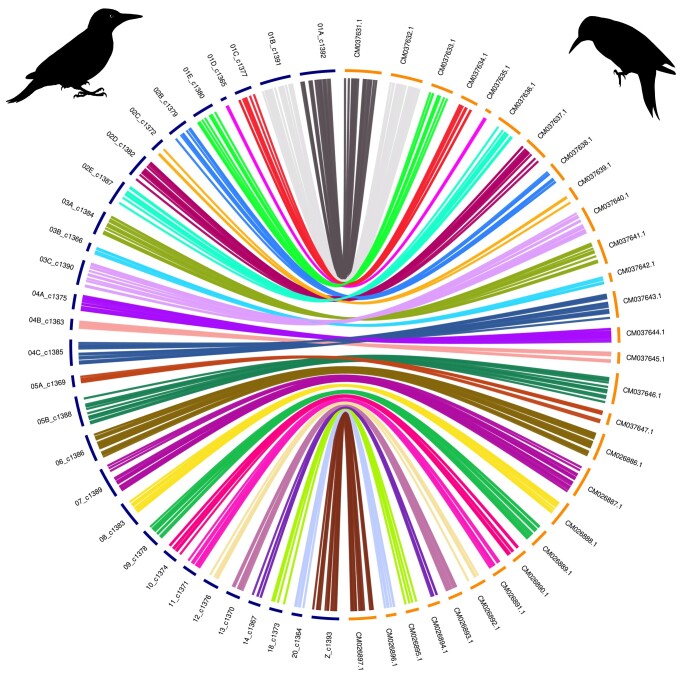
Circos plot. Woodpecker chromosomes are on the left, in blue; Northern flicker (*Colaptes auratus*) chromosomes are on the right, in yellow.

Two circos plots were created to compare the genome assembly produced for *P. viridis* against the chicken, *G. gallus* (Fig. 4) and the Northern Flicker, *C. auratus* (Fig. 5), respectively. *Gallus gallus* was used as a reference because it is commonly employed as a model organism for comparative purposes. Furthermore, their repeat element rate is reflective of that of most avian species (∼10%) (International Chicken Genome Sequencing Consortium 2004). The European green woodpecker genome exhibits higher fragmentation than that of the chicken and is more similar to the genome of *C. auratus* in terms of fragmentation, with an abundance of microchromosomes. This similarity is noteworthy and may account for the difficulties encountered in identifying expected genes in databases such as OrthoDB which logically reduces the BUSCO completeness score.

### Nuclear diversity

In the final VCF, variants with coverage depth within the [10; 346] interval were retained, where 346× represents the value above which 90% of reads were observed in the depth distribution.

**Fig. 6.**
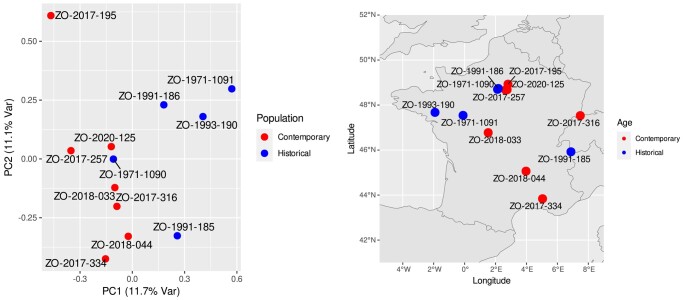
PCA based on genomic diversity among the historical and contemporary samples (left) and map of the sampled *Picus viridis* individiduals (right).

**Fig. 7.**
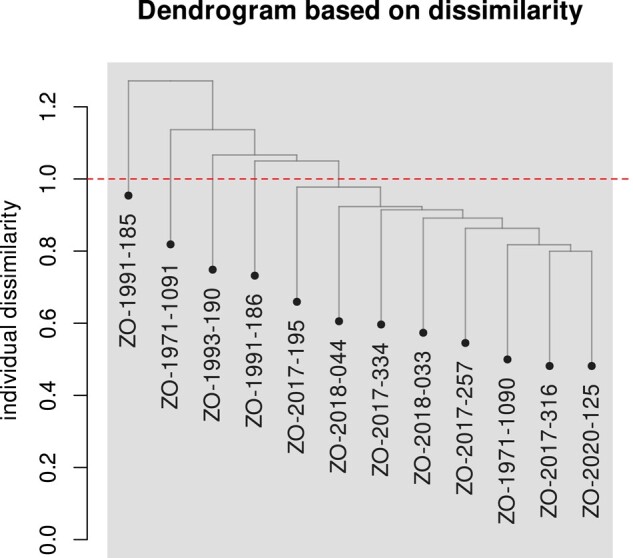
Dendrogram of hierarchical clustering based on pairwise nuclear sequence dissimilarity between samples.

**Fig. 8.**
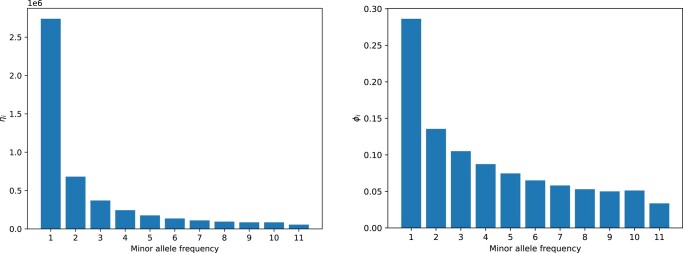
Site frequency spectrum. On left, the raw counts of each allele frequency; on right, the normalized (rescaled [0,1]) and transformed, so that the expected spectrum for a constant population size should be flat at *y* = 1/11.

Before applying depth-based filters, 8,815,726 variants were identified among the 12 individuals. The average depth of variants is 22.7×. We retained 7,226,988 variants that fell within the [10; 346]× range. Higher coverage values are likely caused by repeated sequences. Genetic diversity values were higher in historical specimens than in contemporaneous. The Z chromosome was comparatively more diverse than autosomes (Table 2). On both the PCA (Fig. 6) and the hierarchical clustering dendrogram (Fig. 7), based on sequence dissimilarities, historical and contemporaneous individuals do appear to cluster into 2 groups. These 2 groups can be formed by cutting the dendrogram at a dissimilarity value of 1.0. On the PCA, historical individuals appear slightly more genetically diverse. One historical individual (ZO-1971-1090) is however nested among the contemporary individuals. Frequency spectra (Fig. 8) suggest that the population does not have a constant panmictic demographic history. This pattern may be due to changes in population structure and/or significant changes in population size in the past that are compatible with an expanding population, characterized by an excess of low-frequency variants.

### Mitogenome diversity

We obtained circularized assemblies for 5 out of the 12 samples; we observed length variation among the mitochondrial genomes assembled by Novoplasty, mostly due to the number of a 64 bp repeat at the end of the first control region (which varies between 2 and 5). Two regions were difficult to handle by the algorithm using the shorts reads: the aforementioned 64 bp region located in control region 1 as well as 1 monomeric region in 16S that consists of up to 37°C. Similar problems were found with the BWA mapping algorithm, although the latter tended to provided short genomes (the 64 bp repeat being usually considered as a single repeat with an increase in estimated coverage). We did not have any evidence of nuclear copies of mitochondrial sequences in our mitochondrial assemblies. In a few individuals, heterozygous sites were found in the control region but these sites were in, or bordering, the region with the 64 bp repeats suggesting that misassembly in that particular region is the best hypothesis; the fact that this region had in some cases higher coverage than the remaining parts of the mitochondrial genome gives further credence to this hypothesis. One historical individual (ZO 1993-190) had an R in 16S; such an isolated heterozygosity could also be explained by heteroplasmy.

Prior to the analyses, we excluded 3 regions (positions 966–977 in 12S, position 1,538–1,574 in 16S and 16,023–16,524 in control region) that either consist of monomeric C regions (position 966–977 and 1538–1574) or of the 64 bp region with uncertain number of repeats, resulting in a final alignment of 16,769 bp.

Summary statistics (number of haplotypes H, haplotype diversity Hd, number of segregating sites S, nucleotide diversity π, Watterson's theta Θ), as estimated by DNAsp 5 (Librado and Rozas 2009), are reported in Table 3. The nuclear data and the genetic diversity values are higher in historical specimens than in contemporaneous specimens. The minimum spanning network, as estimated by Pegas (Paradis 2010), is shown in Supplementary Fig. 1. The average *P*-distance among individuals is 0.11% (minimum: 0.005% between two contemporary individuals sampled 400 km apart and 0.17% between a contemporary and historical individual sampled 400 km apart as well).

**Table 3.** Summary statistics for the mitochondrial data, as estimated using DNAsp 5 (Librado and Rozas 2009).

	All	Historical (*n* = 5)	Contemporary (*n* = 7)
H	12	5	7
Hd	1	1	1
S	87	44	48
π	0.00113	0.00115	0.00104
Θ	0.00172	0.00126	0.00117

## Discussion

Using a combination of short and long reads as well as Hi-C data, we successfully assembled a reference genome for a species that had no existing reference genome. The final genome assembly of European Green Woodpecker is estimated to be about 1.28 Gbp. Exhibiting an N50 contig length of 37 Mbp, our assembly displays significant contig continuity, retaining almost all genetic information across the 47 contiguous scaffolds. The de novo assembly and annotation of the *P. viridis* genome presented here represent an important resource for the ornithological community and complements our understanding of the genetic structure of the European green woodpecker. However, it is important to acknowledge that challenges persist with any genome assembly (Peona et al. 2018; Weissensteiner and Suh 2019). Although continuity and completeness have significantly improved, some genomic regions with high GC content and repetitive elements may still present challenges for accurate assembly (Chen *et al*. 2013; Goldstein *et al*. 2019).

### Genome completeness

The BUSCO completeness score of 89.4% (Table 4) is comparatively low for recently assembled bird genomes, especially considering the methods used (Hi-Ci, long reads, and short reads) when compared to other recently published genomes (e.g. Baudrin *et al*. 2023). Among Piciformes, our assembly ranked third out of four species (*M. aurifrons*, *D. pubescens*, *P. viridis*, *C. auratus*) for which long reads were used. We note, however, that the 2 assemblies with the lowest BUSCO scores (*C. auratus*, *P. viridis*) are part of the Picini subclade, whereas the 2 higher scores belong to the Dendropicini (*D. pubescens*) and Melanerpini (*M. aurifrons*) clades (Shakya *et al*. 2017). Genome fragmentation, especially the number of microchromosomes, could explain this pattern as they could be more difficult to assemble. Yet, karyotypic data indicate that *D. pubescens* (2*n* = 92, Shields 1982), *C. auratus* (2*n* = 90, Shields 1982), *P. viridis* (2*n* = 94; Hammar 1970) have similar number of chromosomes. The karyotype of *M. aurifrons*, although currently unknown is possibly much lower than one of its congener, *M. candidus*, which has a chromosome number of 2*n* = 64 (de Oliveira *et al*. 2017). Woodpeckers, unlike most other birds, are particularly rich in repetitive elements (Zhang *et al.* 2014; Manthey *et al*. 2018). The proportion of the genome that consists of repetitive elements is the highest in the two species (*P. viridis*: 30.1%, *C. auratus*: 28%) that have the lower BUSCO scores. It is plausible that the high proportion of repetitive elements in the genomes could also contribute to the lower recovery of BUSCO loci in the assemblies.

**Table 4.** BUSCO analysis to assess the genome completeness.

	De novo assembly (Flye)	Polishing (Pilon)	Hi-C (instaGRAAL)	Hi-C (instaGRAAL) filtered
Total assembly size (bp)	1,278,900,174	1,279,140,189	1,279,164,199	1,262,689,589
Number of contigs	2953	2,953	1,397	47
Average contigs size	433,085.06	501,230.48	915,650.82	26,865,735.94
Largest contig size	23,507,951	23,531,243	107,835,060	107,835,060
N50	4,375,930 (*n* = 82)	4,379,882 (*n* = 82)	36,998,278 (*n* = 12)	36,998,278 (*n* = 12)
Number of gaps	60	2	108	48
Sum of gap size (*N*s count)	6,000	110	24,120	23,310
BUSCO 4.1.4 (aves_odb10)				
BUSCO completeness	7,365 (88.3%)	–	7,457 (89.4%)	7,429 (89.1%)
Single-copy BUSCOs	7,324 (87.8%)	–	7,423 (89.0%)	7,398 (88.7%)
Duplicated BUSCOs	41 (0.5%)	–	34 (0.4%)	31 (0.4%)
Fragmented BUSCOs	462 (5.5%)	–	383 (4.6%)	376 (4.5%)
Missing BUSCOs	511 (6.2%)	–	498 (6.0%)	533 (6.4%)

The analysis was performed 3 times, after significant filtering and assembly steps. Lines 1–7 of the table are given using Assembly Stats (v.1.0.1).

### Spatial structure in historical and contemporaneous data

On the PCA (Fig. 6), there is a visible genomic structure following a north–south gradient on PC2, and a temporal differentiation based on PC1. Indeed the genomic information overall indicates a genetic proximity to birds from nearby regions. Yet, one individual, ZO-2017-195, from the Ile-de-France area, where several modern samples were sequenced, is an outlier on the PCA, although it is located more internally in the dendrogram (Fig. 7). Temporal genetic structuring may exist, where current (expanding) populations are the result of expansion of only a subset of the 1970–1980 population, with potential replacement. Indeed, population expansion may not be homogenous and it is conceivable that not all subpopulations contributed equally to the strong population expansion documented in the last 50 years. Population genomic studies from different time periods will be needed to test this hypothesis.

## Supplementary Material

jkae042_Supplementary_Data

## Data Availability

The PicVir_MNHN_1.0 assembly can be accessed at NCBI (BioProject PRJNA1027323; Genome GCA_033816785.1). All of the related raw sequencing data, specifically Illumina, Nanopore, and Hi-C, can be obtained through NCBI SRA under the same BioProject. Scripts, associated files for this project can be found on GitHub (https://github.com/tforest/P_viridis_assembly). Supplementary files containing data from BUSCO, BRAKER, outputs of MUMmer alignments, genome annotation in GFF format, the VCF file and Supplementary material are available on figshare: https://doi.org/10.6084/m9.figshare.24799065. Supplemental material is available at G3 online.

## References

[jkae042-B1] Achaz G . 2009. Frequency spectrum neutrality tests: one for all and all for one. Genetics. 183(1):249–258. doi:10.1534/genetics.109.104042. 19546320 PMC2746149

[jkae042-B2] Baudrin G , Pons JM, Bed’Hom B, Gil L, Boyer R, Dusabyinema Y, Jiguet F, Fuchs J. 2023. A reference genome assembly for the spotted flycatcher (*Muscicapa striata*). Genome Biol Evol. 15(8):evad140. doi:10.1093/gbe/evad140. 37506263 PMC10402868

[jkae042-B3] Baudry L , Guiglielmoni N, Marie-Nelly H, Cormier A, Marbouty M, Avia K, Mie YL, Godfroy O, Sterck L, Cock JM, et al 2020. instaGRAAL: chromosome-level quality scaffolding of genomes using a proximity ligation-based scaffolder. Genome Biol. 21(1):148. doi:10.1186/s13059-020-02041-z. 32552806 PMC7386250

[jkae042-B4] Birdlife International . 2024. Eurasian Green Woodpecker *Picus viridis*. [accessed 2024 Mar 4]. https://datazone.birdlife.org/species/factsheet/eurasian-greenwoodpecker-picus-viridis.

[jkae042-B5] Castresana J . 2000. Selection of conserved blocks from multiple alignments for their use in phylogenetic analysis. Mol Biol Evol. 17(4):540–552. doi:10.1093/oxfordjournals.molbev.a026334. 10742046

[jkae042-B6] Chen Y-C , Liu T, Yu C-H, Chiang T-Y, Hwang C-C. 2013. Effects of GC bias in next-generation-sequencing data on De Novo genome assembly. PLoS One. 8(4):e62856. doi:10.1371/journal.pone.0062856. 23638157 PMC3639258

[jkae042-B7] Dainat J . 2023. AGAT: another Gff analysis toolkit to handle annotations in any GTF/GFF format. Version v0.7.0. Zenodo. doi:10.5281/zenodo.3552717.

[jkae042-B8] Danecek P , Auton A, Abecasis G, Albers CA, Banks E, DePristo MA, Handsaker RE, Lunter G, Marth GT, Sherry ST, et al 2011. The variant call format and VCFtools. Bioinformatics. 27(15):2156–2158. doi:10.1093/bioinformatics/btr330. 21653522 PMC3137218

[jkae042-B9] de Oliveira TD , Kretschmer R, Bertocchi NA, Degrandi TM, de Oliveira EH, Cioffi MB, Garnero AD, Gunski RJ. 2017. Genomic organization of repetitive DNA in woodpeckers (Aves, Piciformes): implications for karyotype and ZW sex chromosome differentiation. PLoS One. 12(1):e0169987. doi:10.1371/journal.pone.0169987. 28081238 PMC5230766

[jkae042-B10] Dierckxsens N , Mardulyn P, Smits G. 2017. NOVOPlasty: de novo assembly of organelle genomes from whole genome data. Nucleic Acids Res. 45(4):gkw955. doi:10.1093/nar/gkw955. PMC5389512 28204566

[jkae042-B11] Faircloth BC . 2016. PHYLUCE is a software package for the analysis of conserved genomic loci. Bioinformatics. 32(5):786–788. doi:10.1093/bioinformatics/btv646. 26530724

[jkae042-B12] Fu YX . 1995. Statistical properties of segregating sites. Theor Popul Biol. 48(2):172–197. doi:10.1006/tpbi.1995.1025. 7482370

[jkae042-B13] Goldstein S , Beka L, Graf J, Klassen JL. 2019. Evaluation of strategies for the assembly of diverse bacterial genomes using MinION long-read sequencing. BMC Genomics. 20(1):23. doi:10.1186/s12864-018-5381-7. 30626323 PMC6325685

[jkae042-B14] Hammar B . 1970. The karyotypes of thirty-one birds. Hereditas. 65(1):29–58. doi:10.1111/j.1601-5223.1970.tb02306.x.

[jkae042-B15] Hoff KJ , Lange S, Lomsadze A, Borodovsky M, Stanke M. 2016. BRAKER1: unsupervised RNA-Seq-based genome annotation with GeneMark-ET and AUGUSTUS. Bioinformatics. 32(5):767–769. doi:10.1093/bioinformatics/btv661. 26559507 PMC6078167

[jkae042-B16] Hruska JP , Manthey JD. 2021. De novo assembly of a chromosome-scale reference genome for the northern flicker *Colaptes auratus*. G3 (Bethesda). 11(1):jkaa026. doi:10.1093/g3journal/jkaa026. 33561233 PMC8022726

[jkae042-B17] Hu Y , Yan C, Hsu CH, Chen QR, Niu K, Komatsoulis GA, Meerzaman D. 2014. OmicCircos: a simple-to-use R package for the circular visualization of multidimensional omics data. Cancer Inform. 13:13–20. doi:10.4137/CIN.S13495. PMC3921174 24526832

[jkae042-B18] International Chicken Genome Sequencing Consortium . 2004. Sequence and comparative analysis of the chicken genome provide unique perspectives on vertebrate evolution. Nature. 432(7018):695–716. doi:10.1038/nature03154. 15592404

[jkae042-B19] Issa N , Muller Y, Deceuninck B, Pons J-M, Grangé J-L. 2015. Pic vert. Atlas des oiseaux de France métropolitaine: nidification et présence hivernale. Delachaux et Niestlé.

[jkae042-B20] Katoh K , Misawa K, Kuma K, Miyata T. 2002. MAFFT: a novel method for rapid multiple sequence alignment based on fast Fourier transform. Nucleic Acids Res. 30(14):3059–3066. doi:10.1093/nar/gkf436. 12136088 PMC135756

[jkae042-B21] Kolmogorov M , Yuan J, Lin Y, Pevzner PA. 2019. Assembly of long, error-prone reads using repeat graphs. Nat Biotechnol. 37(5):540–546. doi:10.1038/s41587-019-0072-8. 30936562

[jkae042-B22] Kozlov AM , Darriba D, Flouri T, Morel B, Stamatakis A. 2019. RAxML-NG: a fast, scalable and user-friendly tool for maximum likelihood phylogenetic inference. Bioinformatics. 35(21):4453–4455. doi:10.1093/bioinformatics/btz305. 31070718 PMC6821337

[jkae042-B23] Kumar S , Stecher G, Li M, Knyaz C, Tamura K. 2018. MEGA x: molecular evolutionary genetics analysis across computing platforms. Mol Biol Evol. 35(6):1547–1549. doi:10.1093/molbev/msy096. 29722887 PMC5967553

[jkae042-B24] Kurtz S , Phillippy A, Delcher AL, Smoot M, Shumway M, Antonescu C, Salzberg SL. 2004. Versatile and open software for comparing large genomes. Genome Biol. 5(2):R12. doi:10.1186/gb-2004-5-2-r12. 14759262 PMC395750

[jkae042-B25] Librado P , Rozas J. 2009. DnaSP v5: a software for comprehensive analysis of DNA polymorphism data. Bioinformatics. 25(11):1451–1452. doi:10.1093/bioinformatics/btp187. 19346325

[jkae042-B26] Lomsadze A , Burns PD, Borodovsky M. 2014. Integration of mapped RNA-Seq reads into automatic training of eukaryotic gene finding algorithm. Nucleic Acids Res. 42(15):e119. doi:10.1093/nar/gku557. 24990371 PMC4150757

[jkae042-B27] Lomsadze A , Ter-Hovhannisyan V, Chernoff YO, Borodovsky M. 2005. Gene identification in novel eukaryotic genomes by self-training algorithm. Nucleic Acids Res. 33(20):6494–6506. doi:10.1093/nar/gki937. 16314312 PMC1298918

[jkae042-B28] Manthey JD , Moyle RG, Boissinot S. 2018. Multiple and independent phases of transposable element amplification in the genomes of Piciformes (woodpeckers and allies). Genome Biol Evol. 10(6):1445–1456. doi:10.1093/gbe/evy105. 29850797 PMC6007501

[jkae042-B29] Mapleson D , Garcia Accinelli G, Kettleborough G, Wright J, Clavijo BJ. 2017. KAT: a K-mer analysis toolkit to quality control NGS datasets and genome assemblies. Bioinformatics. 33(4):574–576. doi:10.1093/bioinformatics/btw663. 27797770 PMC5408915

[jkae042-B30] Mirchandani CD , Shultz AJ, Thomas GWC, Smith SJ, Baylis M, Arnold B, Corbett-Detig R, Enbody E, Sackton TB. 2024. A fast, reproducible, high-throughput variant calling workflow for population genomics. Mol Biol Evol. 41(1):msad270. doi:10.1093/molbev/msad270. 38069903 PMC10764099

[jkae042-B31] Moreau P , Cournac A, Palumbo GA, Marbouty M, Mortaza S, Thierry A, Cairo S, Lavigne M, Koszul R, Neuveut C. 2018. Tridimensional infiltration of DNA viruses into the host genome shows preferential contact with active chromatin. Nat Commun. 9(1):4268. doi:10.1038/s41467-018-06739-4. 30323189 PMC6189100

[jkae042-B32] Museum national d’Histoire naturelle, Office français de la biodiversité. Picus viridis Linnaeus, 1758—Pic vert, Pivert . Inventaire National du Patrimoine Naturel. https://inpn.mnhn.fr/espece/cd_nom/3603 (accessed 2023 Jul 5).

[jkae042-B33] Nei M , Li WH. 1979. Mathematical model for studying genetic variation in terms of restriction endonucleases. Proc Natl Acad Sci U S A. 76(10):5269–5273. doi:10.1073/pnas.76.10.5269. 291943 PMC413122

[jkae042-B34] Okonechnikov K , Golosova O, Fursov M; UGENE team. 2012. Unipro UGENE: a unified bioinformatics toolkit. Bioinformatics. 28(8):1166–1167. doi:10.1093/bioinformatics/bts091. 22368248

[jkae042-B35] Paradis E . 2010. Pegas: an R package for population genetics with an integrated-modular approach. Bioinformatics. 26(3):419–420. doi:10.1093/bioinformatics/btp696. 20080509

[jkae042-B36] Peona V , Weissensteiner MH, Suh A. 2018. How complete are “complete” genome assemblies?—An avian perspective. Mol Ecol Resour. 18:1188–1195. doi:10.1111/1755-0998.12933. 30035372

[jkae042-B37] Perktas U , Barrowclough GF, Groth JG. 2011. Phylogeography and species limits in the green woodpecker complex (Aves: Picidae): multiple *Pleistocene refugia* and range expansion across Europe and the Near East. Biol J Linnean Soc. 104(3):710–723. doi:10.1111/j.1095-8312.2011.01750.x.

[jkae042-B38] Pfeifer B , Wittelsbürger U, Ramos-Onsins SE, Lercher MJ. 2014. PopGenome: an efficient Swiss army knife for population genomic analyses in R. Mol Biol Evol. 31(7):1929–1936. doi:10.1093/molbev/msu136. 24739305 PMC4069620

[jkae042-B39] Pons J-M , Masson C, Olioso G, Fuchs J. 2019. Gene flow and genetic admixture across a secondary contact zone between two divergent lineages of the Eurasian green woodpecker *Picus viridis*. J Ornithol. 160(4):935–945. doi:10.1007/s10336-019-01675-6.

[jkae042-B40] Pons J-M , Olioso G, Cruaud C, Fuchs J. 2011. Phylogeography of the Eurasian green woodpecker (*Picus viridis*). J Biogeogr. 38(2):311–325. doi:10.1111/j.1365-2699.2010.02401.x.

[jkae042-B41] Privé F , Luu K, Vilhjálmsson BJ, Blum MGB. 2020. Performing highly efficient genome scans for local adaptation with R package pcadapt version 4. Mol Biol Evol. 37(7):2153–2154. doi:10.1093/molbev/msaa053. 32343802

[jkae042-B42] Prum RO , Berv JS, Dornburg A, Field DJ, Townsend JP, Lemmon EM, Lemmon AR. 2015. A comprehensive phylogeny of birds (Aves) using targeted next-generation DNA sequencing. Nature. 526(7574):569–573. doi:10.1038/nature15697. 26444237

[jkae042-B43] Shakya SB , Fuchs J, Pons J-M, Sheldon FH. 2017. Tapping the woodpecker tree for evolutionary insight. Mol Phylogenet Evol. 116:182–191. doi:10.1016/j.ympev.2017.09.005. 28890006

[jkae042-B44] Shields GF . 1982. Comparative avian cytogenetics: a review. Condor. 84(1):45–58. doi:10.2307/1367820.

[jkae042-B45] Simão FA , Waterhouse RM, Ioannidis P, Kriventseva EV, Zdobnov EM. 2015. BUSCO: assessing genome assembly and annotation completeness with single-copy orthologs. Bioinformatics. 31(19):3210–3212. doi:10.1093/bioinformatics/btv351. 26059717

[jkae042-B46] Smit A , Hubley R, Green P. 2013. RepeatMasker Open-4.0. https://www.repeatmasker.org/RepeatMasker/.

[jkae042-B47] Stamatakis A . 2014. RAxML version 8: a tool for phylogenetic analysis and post-analysis of large phylogenies. Bioinformatics. 30(9):1312–1313. doi:10.1093/bioinformatics/btu033. 24451623 PMC3998144

[jkae042-B48] Stanke M , Keller O, Gunduz I, Hayes A, Waack S, Morgenstern B. 2006. AUGUSTUS: ab initio prediction of alternative transcripts. Nucleic Acids Res. 34(Web Server):W435–W439. doi:10.1093/nar/gkl200. 16845043 PMC1538822

[jkae042-B49] Talavera G , Castresana J. 2007. Improvement of phylogenies after removing divergent and ambiguously aligned blocks from protein sequence alignments. Syst Biol. 56(4):564–577. doi:10.1080/10635150701472164. 17654362

[jkae042-B50] Vaser R , Sovic I, Nagarajan N, Sikic M. 2017. Fast and accurate de novo genome assembly from long uncorrected reads. Genome Res. 27:737–746. doi:10.1101/gr.214270.116. 28100585 PMC5411768

[jkae042-B51] Walker BJ , Abeel T, Shea T, Priest M, Abouelliel A, Sakthikumar S, Cuomo CA, Zeng Q, Wortman J, Young SK, et al 2014. Pilon: an integrated tool for comprehensive microbial variant detection and genome assembly improvement. PLoS One. 9(11):e112963. doi:10.1371/journal.pone.0112963. 25409509 PMC4237348

[jkae042-B52] Weissensteiner MH , Suh A. 2019. Repetitive DNA: the dark matter of avian genomics. In: Kraus RHS, editor. Avian Genomics in Ecology and Evolution: From the Lab into the Wild. Cham: Springer International Publishing. p. 93–150.

[jkae042-B53] Wiley G , Miller MJ. 2020. A highly contiguous genome for the golden-fronted woodpecker (*Melanerpes aurifrons*) via hybrid Oxford nanopore and short read assembly. G3 (Bethesda). 10(6):1829–1836. doi:10.1534/g3.120.401059. 32317270 PMC7263694

[jkae042-B54] Winnepenninckx B , Backeljau T, De Wachter R. 1993. Extraction of high molecular weight DNA from molluscs. Trends Genet. 9(12):407. doi:10.1016/0168-9525(93)90102-n. 8122306

[jkae042-B55] Zhang G , Li C, Li Q, Li B, Larkin DM, Lee C, Storz JF, Antunes A, Greenwold MJ, Meredith RW, et al 2014. Comparative genomics reveals insights into avian genome evolution and adaptation. Science. 346(6215):1311–1320. doi:10.1126/science.1251385. 25504712 PMC4390078

[jkae042-B56] Zheng X , Levine D, Shen J, Gogarten SM, Laurie C, Weir BS. 2012. A high-performance computing toolset for relatedness and principal component analysis of SNP data. Bioinformatics. 28(24):3326–3328. doi:10.1093/bioinformatics/bts606. 23060615 PMC3519454

