# Supplementary material for: Chromosome-level genome assembly of the European green woodpecker Picus viridis
Source: G3 (Bethesda). 2024 Mar 27;14(5):jkae042. doi: 10.1093/g3journal/jkae042 (PMC11075563; doi:10.1093/g3journal/jkae042)
Supplement: jkae042_Supplementary_Data [file jkae042_supplementary_data.doc]

**Figure S1.** Mitochondrial minimum Spanning Network built using Pegas (Paradis 2010). Colors refer to historical (blue) and contemporary (red) samples. Number in squares represent the number of mutations.

**
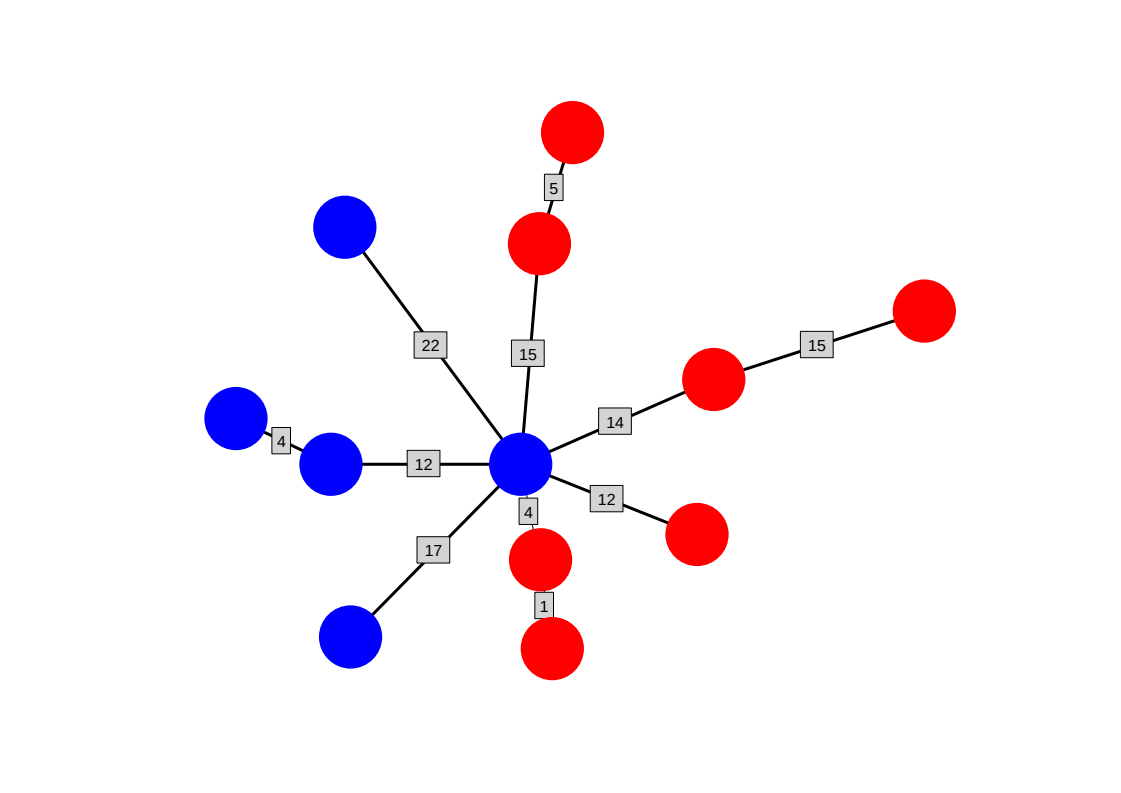
**

### **Table S1.** Sampling scheme for the mitogenome analysis.

| **Family** | **Genera** | **Species** | **GenBank Accession Number** | **Reference** |
| --- | --- | --- | --- | --- |
| Capitonidae | *Capito* | *niger* | MK060126 | Tamashiro et al. (2018) |
| Galbulidae | *Galbula* | *dea* | MN356220 | Feng et al. (2020) |
| Indicatoridae | *Indicator* | *maculatus* | NC039889 | Tamashiro et al. (2018) |
| Indicatoridae | *Indicator* | *maculatus* | MN356234 | Feng et al. (2020) |
| Indicatoridae | *Indicator* | *xanthonotus* | NC040005 | Duan et al. (2018) |
| Indicatoridae | *Prodotiscus* | *insignis* | NC039892 | Tamashiro et al. (2018) |
| Lybiidae | *Pogoniulus* | *bilineatus* | MK060142 | Tamashiro et al. (2018) |
| Lybiidae | *Pogoniulus* | *pusillus* | CM026823 | Kirschel et al. (unpublished) |
| Lybiidae | *Tricholaema* | *leucomelas* | MN356262 | Feng et al. (2020) |
| Megalaimidae | *Psilopogon* | *virens* | NC039891 | Tamashiro et al. (2018) |
| Megalaimidae | *Psilopogon* | *haemacephalus* | NC052781 | Feng et al. (2020) |
| Picidae | *Campephilus* | *guatemalensis* | NC028020 | Fuchs et al. (2016) |
| Picidae | *Campephilus* | *imperialis* | NC034278 | Anmarkrud and Lifjeld JT (2017) |
| Picidae | *Dendrocopos* | *darjellensis* | NC042683 | Bi et al. (2019) |
| Picidae | *Dendrocopos* | *leucotos* | NC029862 | Eo (2017) |
| Picidae | *Dendrocopos* | *major* | NC028174 | Park et al. (2019) |
| Picidae | *Dendrocopos* | *major* | MN122831 | Unpublished |
| Picidae | *Dryobates* | *pubescens* | NC027936 | Zhang et al. (2016) |
| Picidae | *Dryobates* | *minor* | NC061952 | Chen et al. (2022) |
| Picidae | *Dryocopus* | *martius* | LC541437 | Yamamoto et al. (unpublished) |
| Picidae | *Dryocopus* | *pileatus* | NC008546 | Gibb et al. (2007) |
| Picidae | *Dryocopus* | *pileatus* | MK060129 | Tamashiro et al. (2018) |
| Picidae | *Jynx* | *ruficollis* | NC039890 | Tamashiro et al. (2018) |
| Picidae | *Jynx* | *torquilla* | NC056091 | Du et al. (2020) |
| Picidae | *Melanerpes* | *aurifrons* | CM022134 | Wiley and Miller (2020) |
| Picidae | *Picumnus* | *innominatus* | NC039537 | Zhou et al. (2017) |
| Picidae | *Picus* | *canus* | NC045372 | Yao et al. (2019) |
| Picidae | *Picus* | *viridis* | To be added | This study |
| Picidae | *Dendrocopos* | *noguchii* | BJBY01000040.1 | Nakajima et al. (unpublished) |
| Picidae | *Dendrocopos* | *noguchii* | LC541474 | Yamamoto et al. (unpublished) |
| Picidae | *Sasia* | *ochracea* | NC028019 | Fuchs et al. (2016) |
| Picidae | *Yungipicus* | *canicapillus* | NC041121 | Lai et al. (2019) |
| Picidae | *Yungipicus* | *kizuki* | NC062944 | Kim (unpublished) |
| Ramphastidae | *Eubucco* | *bourcierii* | MN356176 | Feng et al. (2020) |
| Ramphastidae | *Pteroglossus* | *azara* | NC008549 | Gibb et al. (2007) |
| Ramphastidae | *Ramphastos* | *sulfuratus* | MN356196 | Feng et al. (2020) |
| Ramphastidae | *Semnornis* | *frantzii* | MN356206 | Feng et al. (2020) |

### **Table S2.** BUSCO scores among six Picidae species.

| **Species** | **Accession number** | **Complete** | **Single-Copy** | **Duplicated** | **Fragmented** | **Missing** | **Reference** |
| --- | --- | --- | --- | --- | --- | --- | --- |
| *Colaptes auratus* | GCA_015227895.2 | 7396 (88.7%) | 7326 (87.9%) | 70 (0.8%) | 279 (3.3%) | 663 (8.0%) | Re-analysis from Hruska and Manthey (2021) |
| *Colaptes auratus* | GCA_015227895.2 | 7294 (87.4%) | 7224 (86.6%) | 70 (0.8%) | 219 (2.6%) | 825 (10.0%) | Hruska and Manthey (2021) |
| *Dendrocopos noguchii* | GCA_004320165.1 | 5255 (63%) | 5237 (62.8%) | 18 (0.2%) | 1449 (17.4%) | 1634 (19.6%) | Re-analysis from Wiley and Miller (2020) |
| *Dryobates pubescens* | GCF_000699005.1 | 7702 (92.4%) | 7681 (92.1%) | 21 (0.3%) | 266 (3.2%) | 370 (4.4%) | Re-analysis from Jarvis et al. (2015) |
| *Melanerpes aurifrons* | GCA_011125475.1 | 7831 (94%) | 7768 (93.2%) | 63 (0.8%) | 204 (2.4%) | 303 (3.6%) | Re-analysis from Wiley and Miller (2020) |
| ***Picus viridis*** | GCA_033816785.1 | 7429 (89.1%) | 7398 (88.7%) | 31 (0.4%) | 376 (5.4%) | 533 (6.4%) | This study |

**Table S3.** Information on sequences used in the UCE analysis

| **Species** | **Accession Number** | **Number of UCE loci retrieved -out of 5041** | **Reference** |
| --- | --- | --- | --- |
| *Galbula dea* | GCA_013399015.1 | 4811 | Feng et al. (2020) |
| ***Picus viridis*** | **GCA_033816785.1** | **4742** | **This study** |
| *Indicator maculatus* | GCA_013399975.1 | 4722 | Feng et al. (2020) |
| *Dendrocopos noguchii* | GCA_004320165.1 | 4707 | Unpublished |
| *Tricholaema leucomelas* | GCA_013400475.1 | 4690 | Feng et al. (2020) |
| *Pogoniulus pusillus* | GCA_015220805.1 | 4684 | Kirschel et al. (2021) |
| *Ramphastos sulfuratus* | GCA_013399055.1 | 4676 | Feng et al. (2020) |
| *Bucco capensis* | GCA_013396975.1 | 4672 | Feng et al. (2020) |
| *Dryobates pubescens* | GCA_000699005.1 | 4664 | Zhang et al. (2014) |
| *Melanerpes aurifrons* | GCA_011125475.1 | 4662 | Wiley and Miller (2020) |
| *Eubucco bourcierii* | GCA_013396675.1 | 4657 | Feng et al. (2020) |
| *Psilopogon haemacephalus* | GCA_013396835.1 | 4571 | Feng et al. (2020) |
| *Colaptes auratus* | GCA_015227895.2 | 4063 | Hruska et a. (2021) |
| *Semnornis frantzii* | GCA_013399775.1 | 4044 | Feng et al. (2020) |

### **Table S4.** Mitochondrial genome as assembled by Novoplasty and BWA. The BWA mapping is not performed for circularized genomes.

| **Sample** | **Length Novoplasty** | **Average Novoplasty Coverage** | **Circularized** | **Average BWA** |
| --- | --- | --- | --- | --- |
| MNHN ZO 1971-1090 | FAIL | FAIL | No | Yes (16932) |
| MNHN ZO 1971-1091 | FAIL | FAIL | No | Yes (16924) |
| MNHN ZO 1991-185 | 17164 | 1151 | Yes | No |
| MNHN ZO 1991-186 | 17296 | 2350 | No | Yes |
| MNHN ZO 1993-190 | 17174 | 878 | Yes | No |
| MNHN ZO 2017-195 | 16972 | 9640 | Yes | No |
| MNHN ZO 2017-257 | 17167 | 24765 | Yes | No |
| MNHN ZO 2017-316 | 17162-17171 | 36094 | No | Yes |
| MNHN ZO 2017-334 | 17188 | 4859 | Yes | No |
| MNHN ZO 2018-033 | 16903 | 85923 | No | Yes |
| MNHN ZO 2018-044 | FAIL | FAIL | No | Yes (16947) |
| MNHN ZO 2020-125 | 16912 | 111722 | No | Yes |

**Table S5.** Statistics for the resequencing nuclear data, after mapping on the assembly using samtools (Danecek et al. 2021) ‘coverage’ command (v1.10)

| **Sample** | **# reads** | **# mapped reads** | **Mean depth (X)** | **Coverage (%)** |
| --- | --- | --- | --- | --- |
| MNHN ZO 1971-1090 | 838522428 | 823494716 | 46.58 | 94.99 |
| MNHN ZO 1971-1091 | 664305384 | 642459019 | 36.28 | 95.11 |
| MNHN ZO 1991-185 | 265383604 | 255255297 | 13.07 | 94.16 |
| MNHN ZO 1991-186 | 479356163 | 460489600 | 24.99 | 94.69 |
| MNHN ZO 1993-190 | 359866383 | 352954499 | 19.82 | 94.40 |
| MNHN ZO 2017-195 | 352341383 | 337387142 | 25.37 | 94.84 |
| MNHN ZO 2017-257 | 361314067 | 348034241 | 23.74 | 94.76 |
| MNHN ZO 2017-316 | 507312904 | 493414745 | 29.32 | 94.68 |
| MNHN ZO 2017-334 | 471819835 | 458378743 | 26.31 | 94.69 |
| MNHN ZO 2018-033 | 376278358 | 357863566 | 25.19 | 94.85 |
| MNHN ZO 2018-044 | 425893410 | 412800708 | 24.04 | 94.76 |
| MNHN ZO 2020-125 | 917559026 | 894729790 | 73.65 | 95.11 |

**Table S6.** BBtools assembly statistics used to compare the assembly of *Picus viridis* in this study with that of the Northern flicker, *Colaptes auratus*.

| **Statistic** | ***Colaptes auratus*** | ***Picus viridis*** |
| --- | --- | --- |
| A | 0.2755 | 0.2727 |
| C | 0.2247 | 0.2271 |
| G | 0.2247 | 0.2272 |
| T | 0.2752 | 0.2729 |
| N | 0.0066 | 0.0000 |
| IUPAC | 0.0000 | 0.0000 |
| Other | 0.0000 | 0.0000 |
| GC | 0.4493 | 0.4544 |
| GC_stdev | 0.0778 | 0.0723 |
| Main genome scaffold total | 2369 | 47 |
| Main genome contig total | 9565 | 49 |
| Main genome scaffold sequence total | 1378.187 MB | 1228.541 MB |
| Main genome contig sequence total | 1369.051 MB (0.663% gap) | 1228.526 MB  (0.001% gap) |
| Main genome scaffold N/L50 | 11/43.948 MB | 11/39.165 MB |
| Main genome contig N/L50 | 281/826.96 KB | 11/36.998 MB |
| Main genome scaffold N/L90 | 33/14.604 MB | 31/17.446 MB |
| Main genome contig N/L90 | 4370/50.09 KB | 32/15.416 MB |
| Max scaffold length | 117.313 MB | 107.835 MB |
| Max contig length | 15.844 MB | 107.832 MB |
| Number of scaffolds > 50 KB | 119 | 47 |
| % main genome in scaffolds > 50 KB | 97.81% | 100.00% |

**Table S7.** Number of missing callable sites for each sample after applying all the filters in the produced VCF, sorted in descending order.

| **Sample** | **Total** |
| --- | --- |
| MNHN ZO 1991-185 | 373 588 |
| MNHN ZO 2018-044 | 254 247 |
| MNHN ZO 1993-190 | 239 754 |
| MNHN ZO 1991-186 | 232 290 |
| MNHN ZO 2017-334 | 223 598 |
| MNHN ZO 2020-125 | 211 803 |
| MNHN ZO 2018-033 | 191 683 |
| MNHN ZO 2017-316 | 188 440 |
| MNHN ZO 2017-257 | 181 157 |
| MNHN ZO 1971-1091 | 175 099 |
| MNHN ZO 1971-1090 | 117 540 |
| **Total** | **2 389 199** |
| **Number of unique sites** | **848 963** |

**Table S8.** Summary of the different types of repeated elements identified using RepeatMasker.

|  | Number of elements | Length occupied | Percentage of sequence |
| --- | --- | --- | --- |
|  |  |  |  |
| **Retroelements** | **864793** | **316153701 bp** | **24.72 %** |
| SINEs | 1587 | 163105 bp | 0.01 % |
| Penelope | 0 | 0 bp | 0.00 % |
| LINEs | 833521 | 296643110 bp | 23.19 % |
| CRE/SLACS | 0 | 0 bp | 0.00 % |
| L2/CR1/Rex | 833521 | 296643110 bp | 23.19 % |
| R1/LOA/Jockey | 0 | 0 bp | 0.00 % |
| R2/R4/NeSL | 0 | 0 bp | 0.00 % |
| RTE/Bov-B | 0 | 0 bp | 0.00 % |
| L1/CIN4 | 0 | 0 bp | 0.00 % |
| LTR elements | 29685 | 19347486 bp | 1.51 % |
| BEL/Pao | 0 | 0 bp | 0.00 % |
| Ty1/Copia | 0 | 0 bp | 0.00 % |
| Gypsy/DIRS1 | 310 | 77693 bp | 0.01 % |
| Retroviral | 29375 | 19269793 bp | 1.51 % |
|  |  |  |  |
| DNA transposons | 4953 | 500460 bp | 0.04 % |
| hobo-Activator | 0 | 0 bp | 0.00 % |
| Tc1-IS630-Pogo | 0 | 0 bp | 0.00 % |
| En-Spm | 0 | 0 bp | 0.00 % |
| MuDR-IS905 | 0 | 0 bp | 0.00 % |
| PiggyBac | 0 | 0 bp | 0.00 % |
| Tourist/Harbinger | 827 | 101481 bp | 0.01 % |
| Other (Mirage, P-element, Transib) | 0 | 0 bp | 0.00 % |
|  |  |  |  |
| Rolling-circles | 0 | 0 bp | 0.00 % |
|  |  |  |  |
| Unclassified | 74215 | 36016205 bp | 2.82 % |
|  |  |  |  |
| **Total interspersed repeats** | | **352670366 bp** | **27.57 %** |
|  |  |  |  |
|  |  |  |  |
| Small RNA: | 0 | 0 bp | 0.00 % |
|  |  |  |  |
| **Satellites** | **902** | **358374 bp** | **0.03 %** |
| **Simple repeats** | **322260** | **27250762 bp** | **2.13 %** |
| **Low complexity** | **56105** | **5021407 bp** | **0.39 %** |

**Table S9.** Summary of the different types of annotations identified using AGAT v1.2.0 (Dainat et. al 2023).

| **mRNA** |  |
| --- | --- |
| Number of gene | 15848 |
| Number of mrna | 16212 |
| Number of mrnas with utr both sides | 5 |
| Number of mrnas with at least one utr | 127 |
| Number of cds | 16212 |
| Number of exon | 77423 |
| Number of five_prime_utr | 73 |
| Number of intron | 61274 |
| Number of start_codon | 16119 |
| Number of stop_codon | 16136 |
| Number of three_prime_utr | 59 |
| Number of exon in cds | 77423 |
| Number of exon in five_prime_utr | 73 |
| Number of exon in three_prime_utr | 59 |
| Number of intron in cds | 61211 |
| Number of intron in exon | 61211 |
| Number of intron in intron | 48395 |
| Number gene overlapping | 73 |
| Number of single exon gene | 3315 |
| Number of single exon mrna | 3330 |
| mean mrnas per gene | 1.0 |
| mean cdss per mrna | 1.0 |
| mean exons per mrna | 4.8 |
| mean five_prime_utrs per mrna | 0.0 |
| mean introns per mrna | 3.8 |
| mean start_codons per mrna | 1.0 |
| mean stop_codons per mrna | 1.0 |
| mean three_prime_utrs per mrna | 0.0 |
| mean exons per cds | 4.8 |
| mean exons per five_prime_utr | 1.0 |
| mean exons per three_prime_utr | 1.0 |
| mean introns in cdss per mrna | 3.8 |
| mean introns in exons per mrna | 3.8 |
| mean introns in introns per mrna | 3.0 |
| Total gene length (bp) | 81357234 |
| Total mrna length (bp) | 85778659 |
| Total cds length (bp) | 16509014 |
| Total exon length (bp) | 16797198 |
| Total five_prime_utr length (bp) | 183082 |
| Total intron length (bp) | 69207345 |
| Total start_codon length (bp) | 48357 |
| Total stop_codon length (bp) | 48408 |
| Total three_prime_utr length (bp) | 105102 |
| Total intron length per cds (bp) | 68981461 |
| Total intron length per exon (bp) | 68981461 |
| Total intron length per intron (bp) | 8834961 |
| mean gene length (bp) | 5133 |
| mean mrna length (bp) | 5291 |
| mean cds length (bp) | 1018 |
| mean exon length (bp) | 216 |
| mean five_prime_utr length (bp) | 2507 |
| mean intron length (bp) | 1129 |
| mean start_codon length (bp) | 3 |
| mean stop_codon length (bp) | 3 |
| mean three_prime_utr length (bp) | 1781 |
| mean cds piece length (bp) | 213 |
| mean five_prime_utr piece length (bp) | 2507 |
| mean three_prime_utr piece length (bp) | 1781 |
| mean intron in cds length (bp) | 1126 |
| mean intron in exon length (bp) | 1126 |
| mean intron in intron length (bp) | 182 |
| Longest gene (bp) | 133036 |
| Longest mrna (bp) | 133036 |
| Longest cds (bp) | 27324 |
| Longest exon (bp) | 14959 |
| Longest five_prime_utr (bp) | 9920 |
| Longest intron (bp) | 31065 |
| Longest start_codon (bp) | 3 |
| Longest stop_codon (bp) | 3 |
| Longest three_prime_utr (bp) | 6787 |
| Longest cds piece (bp) | 14959 |
| Longest five_prime_utr piece (bp) | 9920 |
| Longest three_prime_utr piece (bp) | 6787 |
| Longest intron into cds part (bp) | 31065 |
| Longest intron into exon part (bp) | 31065 |
| Longest intron into intron part (bp) | 14959 |
| Shortest gene (bp) | 45 |
| Shortest mrna (bp) | 45 |
| Shortest cds piece (bp) | 3 |
| Shortest five_prime_utr piece (bp) | 31 |
| Shortest three_prime_utr piece (bp) | 5 |
| Shortest intron into cds part (bp) | 28 |
| Shortest intron into exon part (bp) | 28 |
| Shortest intron into intron part (bp) | 7 |
|  | |
| **Without isoforms (& shortest isoforms excluded)** | |
| Number of gene | 15848 |
| Number of mrna | 15848 |
| Number of mrnas with utr both sides | 5 |
| Number of mrnas with at least one utr | 126 |
| Number of cds | 15848 |
| Number of exon | 72714 |
| Number of five_prime_utr | 73 |
| Number of intron | 56928 |
| Number of start_codon | 15755 |
| Number of stop_codon | 15773 |
| Number of three_prime_utr | 58 |
| Number of exon in cds | 72714 |
| Number of exon in five_prime_utr | 73 |
| Number of exon in three_prime_utr | 58 |
| Number of intron in cds | 56866 |
| Number of intron in exon | 56866 |
| Number of intron in intron | 44398 |
| Number gene overlapping | 72 |
| Number of single exon gene | 3315 |
| Number of single exon mrna | 3315 |
| mean mrnas per gene | 1.0 |
| mean cdss per mrna | 1.0 |
| mean exons per mrna | 4.6 |
| mean five_prime_utrs per mrna | 0.0 |
| mean introns per mrna | 3.6 |
| mean start_codons per mrna | 1.0 |
| mean stop_codons per mrna | 1.0 |
| mean three_prime_utrs per mrna | 0.0 |
| mean exons per cds | 4.6 |
| mean exons per five_prime_utr | 1.0 |
| mean exons per three_prime_utr | 1.0 |
| mean introns in cdss per mrna | 3.6 |
| mean introns in exons per mrna | 3.6 |
| mean introns in introns per mrna | 2.8 |
| Total gene length (bp) | 81357234 |
| Total mrna length (bp) | 81290235 |
| Total cds length (bp) | 15879168 |
| Total exon length (bp) | 16164772 |
| Total five_prime_utr length (bp) | 183082 |
| Total intron length (bp) | 65348767 |
| Total start_codon length (bp) | 47265 |
| Total stop_codon length (bp) | 47319 |
| Total three_prime_utr length (bp) | 102522 |
| Total intron length per cds (bp) | 65125463 |
| Total intron length per exon (bp) | 65125463 |
| Total intron length per intron (bp) | 8321123 |
| mean gene length (bp) | 5133 |
| mean mrna length (bp) | 5129 |
| mean cds length (bp) | 1001 |
| mean exon length (bp) | 222 |
| mean five_prime_utr length (bp) | 2507 |
| mean intron length (bp) | 1147 |
| mean start_codon length (bp) | 3 |
| mean stop_codon length (bp) | 3 |
| mean three_prime_utr length (bp) | 1767 |
| mean cds piece length (bp) | 218 |
| mean five_prime_utr piece length (bp) | 2507 |
| mean three_prime_utr piece length (bp) | 1767 |
| mean intron in cds length (bp) | 1145 |
| mean intron in exon length (bp) | 1145 |
| mean intron in intron length (bp) | 187 |
| Longest gene (bp) | 133036 |
| Longest mrna (bp) | 133036 |
| Longest cds (bp) | 27324 |
| Longest exon (bp) | 14959 |
| Longest five_prime_utr (bp) | 9920 |
| Longest intron (bp) | 31065 |
| Longest start_codon (bp) | 3 |
| Longest stop_codon (bp) | 3 |
| Longest three_prime_utr (bp) | 6787 |
| Longest cds piece (bp) | 14959 |
| Longest five_prime_utr piece (bp) | 9920 |
| Longest three_prime_utr piece (bp) | 6787 |
| Longest intron into cds part (bp) | 31065 |
| Longest intron into exon part (bp) | 31065 |
| Longest intron into intron part (bp) | 14959 |
| Shortest gene (bp) | 45 |
| Shortest mrna (bp) | 45 |
| Shortest cds piece (bp) | 3 |
| Shortest five_prime_utr piece (bp) | 31 |
| Shortest three_prime_utr piece (bp) | 5 |
| Shortest intron into cds part (bp) | 28 |
| Shortest intron into exon part (bp) | 28 |
| Shortest intron into intron part (bp) | 7 |
|  |  |
| **Transcript** | |
| Number of gene | 15909 |
| Number of transcript | 15995 |
| Number gene overlapping | 0 |
| Number of single exon gene | 15909 |
| mean transcripts per gene | 1.0 |
| Total gene length (bp) | 87223346 |
| Total transcript length (bp) | 82809862 |
| mean gene length (bp) | 5482 |
| mean transcript length (bp) | 5177 |
| Longest gene (bp) | 5239356 |
| Longest transcript (bp) | 133036 |
| Shortest gene (bp) | 45 |
| Shortest transcript (bp) | 45 |

**References cited in Supplementary Material**

Anmarkrud JA, Lifjeld JT. 2017a. Complete mitochondrial genomes of eleven extinct or possibly extinct bird species. Mol Ecol Resour. 17:334–341. doi: 10.1111/1755-0998.12600.

Bi D et al. 2019. Two new mitogenomes of Picidae (Aves, Piciformes): Sequence, structure and phylogenetic analyses. International Journal of Biological Macromolecules. 133:683–692. doi: 10.1016/j.ijbiomac.2019.04.157.

Chen J et al. 2022. Characterization of the complete mitochondrial genome of the Lesser Spotted Woodpecker (Dryobates minor) and its phylogenetic position. Mitochondrial DNA Part B. 7:1504–1506. doi: 10.1080/23802359.2022.2110530.

Dainat, J., D. Hereñú, K. D. Murray, E. Davis, K. Crouch *et al.*, 2023 AGAT: Another Gff Analysis Toolkit to handle annotations in any GTF/GFF format. Zenodo. https://www.doi.org/10.5281/zenodo.3552717

Danecek, P., A. Auton, G. Abecasis, C. A. Albers, E. Banks *et al.*, 2011 The variant call format and VCFtools. Bioinformatics 27: 2156–2158.

Du C, Liu LI, Liu Y, Fu Z. 2020. The complete mitochondrial genome of the Eurasian wryneck Jynx torquilla (Aves: Piciformes: Picidae) and its phylogenetic inference. Zootaxa. 4810:zootaxa.4810.2.8. doi: 10.11646/zootaxa.4810.2.8.

Duan Y, Li Y, Liang D, Shao S, Luo X. 2018. Complete mitochondrial genome of yellow-rumped honeyguide Indicator xanthonotus (Piciformes: Indicatoridae). Mitochondrial DNA Part B. 3:1278–1279. doi: 10.1080/23802359.2018.1532837.

Eo SH. 2017. Complete mitochondrial genome of white-backed woodpecker Dendrocopos leucotos (Piciformes: Picidae) and its phylogenetic position. Mitochondrial DNA Part B. 2:451–452. doi: 10.1080/23802359.2017.1357454.

Feng S et al. 2020. Dense sampling of bird diversity increases power of comparative genomics. Nature. 587:252–257. doi: 10.1038/s41586-020-2873-9.

Fuchs J, Pons J-M, Pasquet E, Bonillo C. 2016. Complete mitochondrial genomes of the white-browed piculet (Sasia ochracea, Picidae) and pale-billed woodpecker (Campephilus guatemalensis, Picidae). Mitochondrial DNA A DNA Mapp Seq Anal. 27:3640–3641. doi: 10.3109/19401736.2015.1079834.

Gibb GC, Kardailsky O, Kimball RT, Braun EL, Penny D. 2007. Mitochondrial genomes and avian phylogeny: complex characters and resolvability without explosive radiations. Mol Biol Evol. 24:269–280. doi: 10.1093/molbev/msl158.

Hruska JP, Manthey JD. 2021. De novo assembly of a chromosome-scale reference genome for the northern flicker Colaptes auratus. G3 (Bethesda). 11:jkaa026. doi: 10.1093/g3journal/jkaa026.

Jarvis ED et al. 2015. Phylogenomic analyses data of the avian phylogenomics project. Gigascience. 4:4. doi: 10.1186/s13742-014-0038-1.

Kirschel ANG et al. 2021. Taxonomic revision of the Red-fronted Tinkerbird Pogoniulus pusillus (Dumont, 1816) based on molecular and phenotypic analyses. bbrc. 141:428–442. doi: 10.25226/bboc.v141i4.2021.a6.

Lai W-N, Yan S-Q, Jiao S-Y, Yao J-Y, Li Y-M. 2019. Complete mitochondrial genome of Dendrocopos canicapillus (Piciformes: Picidae). Mitochondrial DNA Part B. 4:141–142. doi: 10.1080/23802359.2018.1544051.

Paradis E. 2010. pegas: an R package for population genetics with an integrated-modular approach. Bioinformatics. 26:419–420. doi: 10.1093/bioinformatics/btp696.

Park CE et al. 2019. The complete mitochondrial genome sequence of Dendrocopos major (Aves, Piciformes, Picidae). Mitochondrial DNA Part B. 4:777–778. doi: 10.1080/23802359.2019.1565980.

Tamashiro RA et al. 2018. What are the roles of taxon sampling and model fit in tests of cyto-nuclear discordance using avian mitogenomic data? Mol Phylogenet Evol. 130:132–142. doi: 10.1016/j.ympev.2018.10.008.

Wiley G, Miller MJ. 2020. A Highly Contiguous Genome for the Golden-Fronted Woodpecker (Melanerpes aurifrons) via Hybrid Oxford Nanopore and Short Read Assembly. G3 (Bethesda). 10:1829–1836. doi: 10.1534/g3.120.401059.

Yao J-Y et al. 2019. The complete mitochondrial genome of Picus canus (Piciformes: Picidae). Mitochondrial DNA Part B. 4:1869–1870. doi: 10.1080/23802359.2019.1614887.

Zhang G et al. 2014. Comparative genomics reveals insights into avian genome evolution and adaptation. Science. 346:1311–1320. doi: 10.1126/science.1251385.

Zhang Z, An M, Deng Y, Zhu S. 2016. The complete mitochondrial genome of the Downy woodpecker, Picoides pubescens (Piciformes: Picidae). Mitochondrial DNA A DNA Mapp Seq Anal. 27:3479–3480. doi: 10.3109/19401736.2015.1066357.

Zhou C et al. 2017. The first complete mitogenome of Picumnus innominatus (Aves, Piciformes, Picidae) and phylogenetic inference within the Picidae. Biochemical Systematics and Ecology. 70:274–282. doi: 10.1016/j.bse.2016.12.003.
